# Evaluation of Six Novel Protein Sources on Apparent Digestibility in Pacific White Shrimp, *Litopenaeus vannamei*

**DOI:** 10.1155/2022/8225273

**Published:** 2022-11-02

**Authors:** Xiaoyue Li, Yongkang Chen, Chaozhong Zheng, Shuyan Chi, Shuang Zhang, Beiping Tan, Shiwei Xie

**Affiliations:** ^1^Laboratory of Aquatic Nutrition and Feed, College of Fisheries, Guangdong Ocean University, Zhanjiang 524088, China; ^2^Guangdong Provincial Key Laboratory of Improved Variety Reproduction in Aquatic Economic Animals, Institute of Aquatic Economic Animals, School of Life Sciences, Sun Yat-sen University, Guangzhou 510275, China; ^3^Aquatic Animals Precision Nutrition and High-Efficiency Feed Engineering Research Centre of Guangdong Province, Zhanjiang 524088, China; ^4^Key Laboratory of Aquatic, Livestock and Poultry Feed Science and Technology in South China, Ministry of Agriculture, Zhanjiang 524088, China

## Abstract

This study is aimed at evaluating the apparent digestibility coefficients (ADC) of six novel protein sources in Pacific white shrimp (*Litopenaeus vannamei*), including black soldier fly larvae meal (BSFLM), *Chlorella vulgaris* meal (CM), cottonseed protein concentrate (CPC), *Tenebrio molitor* meal (TM), *Clostridium autoethanogenum* protein (CAP), and methanotroph (*Methylococcus capsulatus*, Bath) bacteria meal (BPM). The control diet (CD) was formulated to contain 448.8 g/kg crude protein and 71.8 g/kg crude lipid. Then, six experimental diets were formulated to contain 70% CD and 30% test ingredients. The yttrium oxide was used as an exogenous indicator for apparent digestibility detection. Six hundred and thirty healthy and uniform-sized shrimp (approximately 3.04 ± 0.01 g) were randomly distributed into triplicate groups of 30 shrimp and they were fed three times daily. After the shrimp was acclimating for one week, their feces were collected 2 hours after the morning feeding until sufficient samples were available for compositional analysis to calculate apparent digestibility. The apparent digestibility coefficients for a dry matter of diets (ADC_D_) and ingredients (ADC_I_) as well as the apparent digestibility coefficients for crude protein (ADC_Pro_), crude lipid (ADC_L_), and phosphorus (ADC_P_) of test ingredients were calculated. Results showed that the growth performance of shrimp fed BSFLM, TM, and BPM diets significantly decreased compared to that fed the CD (*P* < 0.05), and no significant differences were found among those fed CD, CM, CAP, and CPC diets (*P* > 0.05). There were no significant differences in survival among each group (*P* > 0.05). As for the diets, results showed that the ADC_D_ of BSFLM, CM, CPC, and TM diets was significantly lower than that of CD, while that of the CAP diet was significantly higher than that of CD (*P* < 0.05) and there were no significant differences between BPM and CD diets (*P* > 0.05). As for the test ingredients, the ADC_Pro_ and ADC_L_ of BSFLM, CM, CPC, and TM were significantly lower than those of CD in *Litopenaeus vannamei* (*P* < 0.05). The ADC_Pro_ of CAP was significantly higher than that of CD (*P* < 0.05), but no significant differences were found in ADC_L_ between CAP and CD (*P* > 0.05). The ADC_Pro_ of BPM was significantly lower than that of CD (*P* < 0.05), but there were no significant differences in ADC_L_ between BPM and CD (*P* > 0.05). The ADC_P_ of CM, CAP, and BPM were significantly higher than that of CD, while that of BSFLM was significantly lower than that of CD (*P* < 0.05), and no significant differences were found in ADC_P_ between TM and CD (*P* > 0.05). To conclude, newly developed protein sources such as single-cell protein (CAP, BPM, and CM) showed great potential as a fishmeal alternative, and insect protein meals (TM and BSFLM) were less effective for shrimp compared to the CD. Although the utilization of CPC by shrimp was lower than other protein sources, it had been much improved compared to the untreated cottonseed meal. The present study will contribute to the application of novel protein sources in shrimp feeds.

## 1. Introduction

The production of the Pacific white shrimp, *Litopenaeus vannamei*, reached 4.9 million tonnes in 2018, accounting for 52.9% of the crustacean production and 4.3% of the total aquaculture production in the world, making it one of the most important traded aquatic species globally (data from https://www.fao.org/fishery/en/collection/aquaculture?lang=en). The increasing shrimp production has stimulated the demand for shrimp feed. In terms of nutrients, the crude protein content accounts for 25%-50% of shrimp feed and it is also one of the most expensive constitutions [1]. And fishmeal (FM) has been considered the key ingredient in the feed due to its high protein content, balanced amino acid profile, high digestibility, and good palatability [2] and usually makes up 15%-35% of shrimp feed [3]. However, FM production has been stagnant during the last decades, and the catches used for FM production have decreased due to the El Niño phenomenon [4]. Therefore, the development of alternative protein sources has become an urgent issue to address and studies involving fishmeal alternatives are being carried out [5].

To assess a novel protein source, apparent digestibility is an important indicator for nutrient digestibility and absorption in animals [6]. High apparent digestibility can not only reduce the feed coefficients but also decrease the pollution of the water environment [7]. In this study, the apparent digestibility of six novel protein sources was evaluated, including black soldier fly (*Hermetia illucens*) larvae meal (BSFLM), *Chlorella vulgaris* meal (CM), cottonseed protein concentrate (CPC), *Tenebrio molitor* meal (TM), *Clostridium autoethanogenum* protein (CAP), and methanotroph (*Methylococcus capsulatus*, Bath) bacteria meal (BPM). The black soldier fly larvae are usually fed on waste organic matter such as kitchen waste and livestock manure. Based on high bioconversion capacity, black soldier fly larvae can convert vast amounts of organic waste into their biomass, which can be used for commercial solutions to environmental problems associated with manure and other organic waste [8]. On account of the abundant nutrient value, the black soldier fly larvae meal is currently studied as feed ingredients [9, 10]. In a previous study, dietary BSFLM reduced lipid digestibility but had no effects on the apparent digestibility of protein and dry matter in rainbow trout (*Oncorhynchus mykiss*) [11]. Also, the activities of lipase and amylase in the intestine of grass carp (*Ctenopharyngodon idella*) were significantly reduced after more than 50% of soybean meal was replaced with BSFLM in the feed [12]. These results indicated a decreasing trend in digestibility after feeding with dietary BSFLM. Another insect species, *Tenebrio molitor*, is initially considered a storage pest. However, due to its high nutrient value (crude protein 47%-60%), it is also studied as feed ingredients in pets, livestock, and aquatic animals [13]. Nevertheless, the effects of dietary TM on the digestibility of different animals were inconsistent. For example, the digestibility of protein, lipid, and dry matter was significantly reduced in gilthead sea bream (*Sparus aurata*) when fed with dietary TM, while these digestibility indicators were slightly improved in European sea bass (*Dicentrarchus labrax* L.) [14, 15].

Single-cell protein (SCP) is a mixture obtained from the cytoplasm of algae, yeast, or bacteria, with the advantages of high production efficiency, wide sources of production materials, land saving, and less influence by seasonal and climatic changes [16]. Generally, SCP contains high protein content (30%-50% in yeasts, 40%-70% in microalgae, and 50%-80% in bacteria), carbohydrates, nucleic acids, polyunsaturated fatty acids, minerals, and vitamins. *Chlorella vulgaris* is a unicellular microalga belonging to the *Chlorellaceae* family that can grow in autotrophic and heterotrophic conditions. Chlorella meal normally contains 50%-60% crude protein and 15%-22% crude lipid [17]. In addition, microalgae are rich in astaxanthin, which can be added to the feed to effectively improve the color of shrimp and increase their antioxidant capacity and resistance to stress during harvest and transport [18]. It is still unclear how dietary CM affects digestibility in shrimp, and inconsistent results have been reported [19, 20]. The production of bacterial protein meal is less dependent on land, water, and climate conditions than CM and has high production efficiency and pure nutritional value [21]. Research on BPM and CAP is gaining popularity, and both are showing good application prospects. Methane-oxidizing bacteria (*Methylococcus capsulatus*) are gram-negative bacteria capable of producing BPM by fermentation using methane as the carbon source, and the BPM products usually contain 71% crude protein and 8% crude lipid [22]. In previous studies, the digestibility significantly decreased when 40% of fishmeal was replaced with BPM in turbot juveniles (*Scophthalmus maximus* L.), while no significant differences were found in Japanese yellowtail (*Seriola quinqueradiata*) [23, 24]. *Clostridium autoethanogenum*, an anaerobic gram-positive bacterium, can produce both ethanol and protein byproducts by consuming carbon monoxide from steelmaking converter gas as the carbon source through gas pretreatment, fermentation, distillation, filtration, and spray drying steps [25]. The CAP contains more than 80% crude protein, is rich in lysine, and can replace 30% of fishmeal in the feed without affecting the growth performance of *Litopenaeus vannamei* [26]. Several studies demonstrated that dietary CAP does not affect digestibility at low levels of inclusion, but high inclusion levels may reduce digestibility in animals [27, 28]. The CPC is a protein concentrate product obtained through low-temperature leaching and solvent extraction of traditional cottonseed meal, which has lower cotton phenol content and higher protein content (60%-70%) than cottonseed meal, and is a more ideal substitute for FM [29, 30]. Since few studies have evaluated the digestibility of these six novel protein sources in Pacific white shrimp, in this study, apparent digestibility was determined by the exogenous indicator method to provide valid data to support better utilization of novel protein sources in Pacific white shrimp feed.

## 2. Materials and Methods

### 2.1. Preparation of Experimental Diets

The control diet (CD) was formulated according to the nutritional requirements of the shrimp [31], containing 448.8 g/kg crude protein and 71.8 g/kg crude lipid. As shown in Table 1, the experimental feed was formulated to contain 70% of CD and 30% of test ingredients, and yttrium trioxide was added as an exogenous indicator. All ingredients were sieved through an 80-mesh screen, weighed accurately, and configured into a homogeneous mixture in accordance with the step-by-step amplification. After being sieved through a 60-mesh screen, the mixture was extruded into 1.0 mm diameter pellets using a pelletizer (Institute of Chemical Engineering, South China University of Technology, Guangdong, China) and then ripened in an electric oven at 60°C for 30 min and stored at -20°C before use. The nutrient level and amino acid composition of the ingredients are shown in Table 2.

### 2.2. Shrimp Feeding and Management

The experimental shrimp were purchased from Guangdong Haida Group (Zhanjiang) and fed with commercial feed for two months. The formal experiment was conducted in an indoor seawater culture system; 630 healthy and uniform-sized shrimp (initial weight 3.04 ± 0.01 g) were randomly divided into 7 groups, with triplicate fiberglass tanks (300 L) per group and 30 shrimp in each tank. Shrimp were fed three times a day at 7:00, 12:00, and 20:00 with 6%-8% of body weight per day. During the experiment, 50% of seawater was changed every day. The water temperature was 25-28°C and the salinity was 25-30‰.

### 2.3. Feces Collection

Feces collection was conducted after a one-week feeding trial. To be specific, the residual feed was cleaned up 0.5 h after morning feeding. The fresh feces were collected from each tank by siphoning 2 h after feeding. Furthermore, the intact and coated feces were selected and stored in a sterile tube at -20°C prior to determination. After sufficient samples were collected, they were dried at 105°C and ground before analysis.

### 2.4. Sample Analysis

After a 4-week feeding trial, shrimp in each tank were counted and weighed. Moisture of diets was determined by oven drying at 105°C: weight reduction of feed after drying. Crude protein of feces and diets was detected by Primacs100 analyzer (Skalar, Dutch): after full combustion of the feed, the nitrogen oxides are reduced to nitrogen (crude protein = Total − *N* × 6.25). Crude lipid was detected by an XT15 extractor (Ankom, USA): weight reduction of feed after extraction by petroleum ether. Ash was detected by burning at 550°C: weight reduction of feed after fully burning [32, 33]. The amino acid compositions of ingredients were determined by an automatic amino acid analyzer 433D (Sykam, Germany) after hydrolysis in 6 M HCl for 24 h at 110°C. After being digested with nitric acid and hydrogen peroxide (6 mL 68% nitric acid and 1 mL 30% hydrogen peroxide) by microwave digestion (Anton Paar Multiwave PRO 41HVT56, Austria), samples were conducted in an inductively coupled plasma-mass spectrometer (ICP-MS, Agilent 7500cx, USA) to determine the phosphorus content. The nutrient levels and amino acid composition of diets are shown in Table 3.

### 2.5. Calculations and Statistical Analysis

The parameters of growth performance were calculated as follows [34]:

(1) $$\begin{array}{c} \text{Weight gain rate }\left(\text{WGR},\%\right)=\frac{\left(\text{final body weight}-\text{initial body weight}\right)}{\text{initial body weight}}\times 100\%,\\ \text{Survival }\left(\%\right)=\left(\frac{\text{final shrimp number}}{\text{initial shrimp number}}\right)\times 100\%,\\ \text{Specific growth rate }\left(\text{SGR},\%{\text{day}}^{-1}\right)=\frac{\left[\ln\left(\text{final body weight}\right)-\ln\left(\text{initial body weight}\right)\right]}{\text{days}}\times 100\%,\\ \text{Feed efficiency }\left(\text{FE}\right)=\frac{\text{feed consumption}}{\text{body weight gain}}. \end{array}$$

The apparent digestibility coefficient (ADC) of dry matter (ADC_D_), ingredients (ADC_I_), crude protein (ADC_Pro_), crude lipid (ADC_L_), phosphorus (ADC_P_), and amino acids (ADC_AA_) were calculated as follows: [35]:

(2) $$\begin{array}{c} {\text{ADC}}_{\text{D}}\left(\%\right)=100\%\times \left[1-\left(\frac{\text{Md}}{\text{Mf}}\right)\right],\\ \text{ADC of nutrients in diets }\left(\%\right)=100\%\times \left(1-\frac{\text{Nf}}{\text{Nd}}\times \frac{\text{Md}}{\text{Mf}}\right), \end{array}$$

where Md and Mf are the percentage of yttrium oxide in diets and feces, respectively, and Nd and Nf are the percentage of nutrient in diets and feces, respectively.

(3) $$\begin{array}{c} \text{ADC of nutrients in ingredients }\left(\%\right)=\text{ADCt}+\left[\left(\text{ADCt}-\text{ADCr}\right)\times \left(\frac{0.7\times \text{Nr}}{0.3\times \text{Ni}}\right)\right], \end{array}$$

where ADCt is the ADC of nutrients in test diets and ADCr is the ADC of nutrients in CD, while Nr and Ni are the nutrient contents of the CD and test diets, respectively.

Data were subjected to one-way analysis of variance (ANOVA) followed by Duncan's test to determine significant differences among treatments using SPSS 21.0 (SPSS, Chicago, IL, USA). Probability value of *P* < 0.05 was deemed to be statistically significant.

## 3. Results

### 3.1. Growth Performance

As shown in Table 4, there were no significant differences in final body weight (FBW), weight gain rate (WGR), and specific growth rate (SGR) of shrimp fed the CD, CM, CPC, and CAP diets (*P* > 0.05). But the FBW, WGR, and SGR of shrimp fed the BSFLM, TM, and BPM diets significantly decreased compared to those fed the CD (*P* < 0.05). There was no significant difference in survival among shrimp fed different diets (*P* > 0.05). The feed efficiency (FE) of shrimp fed the BSFLM diet was significantly higher than that fed the CD (*P* < 0.05). There were no significant differences in FE among the shrimp fed the CM, CPC, TM, CAP, and BPM diets compared to those fed the CD (*P* > 0.05).

### 3.2. Apparent Digestibility of Dry Matter, Ingredients, Crude Protein, Crude Lipid, and Phosphorus

As shown in Table 5, the apparent digestibility coefficients of dry matter (ADC_D_) in diets ranged from 67.52% to 83.46% and the apparent digestibility coefficients of ingredients (ADC_I_) in test ingredients ranged from 39.67% to 97.41%. To be specific, the ADC_D_ of BSFLM, CM, CPC, and TM diets was significantly lower than that of CD, while the ADC_D_ of CAP diets was significantly higher than that of CD (*P* < 0.05). There was no significant difference in ADC_D_ between the CD and BPM diets (*P* > 0.05). The apparent digestibility coefficients of protein (ADC_Pro_) in test ingredients ranged from 56.50% to 97.74%. Briefly, the ADC_Pro_ of BSFLM, CM, CPC, TM, and BPM was significantly lower than that of CD, while ADC_Pro_ of CAP was significantly higher than that of CD (*P* < 0.05). The apparent digestibility coefficients of lipid (ADC_L_) in test ingredients ranged from 70.53% to 94.05%. Briefly, the ADC_L_ of BSFLM, CM, CPC, and TM was significantly lower than that of CD (*P* < 0.05), while there were no significant differences in ADC_L_ among CD, BPM, and CAP (*P* > 0.05). The apparent digestibility coefficients of phosphorus (ADC_P_) in test ingredients ranged from 41.40% to 90.95%. Briefly, the ADC_P_ of BSFLM was significantly lower than that of CD, while ADC_P_ of CM, BPM, and CAP was significantly higher than that of CD (*P* < 0.05). There were no significant differences in ADC_P_ between CD and TM (*P* > 0.05). Due to insufficient samples, the determination of ADC_P_ for CPC could not be conducted, so the corresponding data are not provided.

### 3.3. Apparent Digestibility of Amino Acids

The ADC of amino acids (ADC_AA_) of test ingredients is shown in Table 6. The ADC of all amino acids except histidine of CAP was higher than that of CD. BPM exhibited better ADC of lysine and methionine and the ADC of other amino acids was lower than or similar to that of the CD. The ADC for methionine of CPC was similar to that of CD and that of other amino acids was lower than the CD. The ADC of all amino acids of CM and BSFLM was lower than that of the CD. Compared to the CD, TM exhibited a similar ADC of methionine and the ADC of other amino acids was lower.

## 4. Discussion

Determining the apparent digestibility of ingredients is an important prerequisite for evaluating the availability of novel protein sources [36]. The assessment of ADC_D_ helps to learn the total amount of nutrients being digested, as the components of the feed are not digested by the animal in the same proportions [37]. In the present study, shrimp showed divergent ADC_D_ and ADC_I_ when fed with different test diets, which were closely related to ingredients. For insect protein sources, shrimp fed with dietary BSFLM and TM showed a significantly lower ADC_D_ of diets as well as ADC_Pro_ and ADC_L_ of test ingredients than CD. Also, the ADC_Pro_ and ADC_L_ of BSFLM were significantly higher than those of TM. The digestive properties of shrimp fed with insect protein are strongly influenced by the nutritional properties of the ingredients. Typically, the crude protein, crude lipid, nitrogen-free extracts, and ash content of insect proteins varied with species and growth stage [38]. Furthermore, insect exoskeletons are usually composed of chitin, which is generally considered to impede the digestive process [39, 40]. Previous studies demonstrated that BSFLM (crude protein 30.0%, crude lipid 33.9%) generally contained a higher chitin level than TM (crude protein 42.0%, crude lipid 28.3%), and therefore, rainbow trout (*Oncorhynchus mykiss*) digested TM better than BSFLM [41]. However, no significant differences were found in the apparent digestibility of protein and lipid between two dietary insect proteins (BSFLM, crude protein 36.4%, crude lipid 11.0%; TM, crude protein 38.7%, crude lipid 12.6%) in the Pacific white shrimp [42]. This is probably because 28.0%-35.5% of dietary chitin in insect meals can be digested by shrimp. In the research on TM (crude protein 55.6%, crude lipid 34.6%), the value of ADC was 45.9% for dry matter and 76.1% for ADC_Pro_, and the apparent digestibility of essential amino acids ranged from 72.86% to 86.05% [43], which seems different from our results. This could be explained by the different nutrient compositions of ingredients since the defatted TM may contain higher levels of chitin and the difference in the digestibility system in different growth stages of shrimp. Further, it was observed that the insect protein source had the lowest amino acid digestibility compared to other protein sources in the present study. This can be caused by the chitin being bound to the protein by a covalent bond and negatively affecting their being digested by the shrimp [44]. Although chitin is not easily digested by shrimp, it can act as an immune booster and improve the immune capacity of shrimp [45]. TM and BSFLM have received wide attention due to the great potential for aquafeed application, while the former has been approved by the EU's European Food Safety Authority as a new food [46], and the latter is considered an important biomass resource [47]. A previous study showed that dietary TM contributed a promotional effect to the growth of Mandarin fish (*Siniperca scherzeri*) and rockfish (*Sebastes schlegelii*) [48, 49]. The WGR and FE of the Pacific white shrimp were significantly increased when 50% of FM was replaced with TM [50]. For BSFLM, our previous study demonstrated that the replacement of 20% FM with BSFLM had no negative effects on the growth performance and was beneficial to the gut microbiota composition and lipid metabolism of *Litopenaeus vannamei* [51]. Although the utilization of insect proteins is limited by many reasons, such as the food safety concern of the ingredients and the digestive hindrance of chitin [52], efforts have been made to improve their availability.

Single-cell protein (SCP) is the biomass obtained from the cytoplasm of algae, yeast, bacteria, or fungi, which has attracted attention due to the advantage of nutritional characteristics and high production in the limited reactor [53]. In the present study, the apparent digestibility of CM, BPM, and CAP in Pacific white shrimp was measured. The results showed that most of the apparent digestibility indexes in CM were significantly lower than those in CD, but the ADC_P_ of these three SCP was significantly higher than that in CD. Previous studies showed that the ADC_P_ significantly increased with the increase in bacterial protein meal or microalgae meal [54], indicating a higher effective phosphorus content in SCPs. As a natural food for marine organisms, microalgae appear to be easily utilized in shrimp diets [55]. A previous study showed that dietary CM can replace 75% FM in shrimp diet without negative effects on growth performance, but the digestive enzyme activities including trypsin, chymotrypsin, and amylase decreased at high levels of CM inclusion [56]. Microalgae generally contain more than 400 g/kg of carbohydrates, a large proportion of which consists of complex and structural carbohydrates (e.g., crude fiber), which may cause negative effects on the digestibility of shrimp [57]. On the other hand, the pretreatment process of SCP also affects the digestive characteristics of animals. A previous study showed that dietary whole-cell CM may reduce the digestibility of dry matter, protein, and lipid, while the cell-ruptured CM did not negatively affect the ADC_D_ and ADC_Pro_ of Atlantic salmon (*Salmo salar* L.) [20]. Therefore, rupture of SCP cell wall by physical, chemical hydrolysis, or bioenzymatic methods often releases more protein and amino acid profiles and reduces the antinutrition factors, making intracellular nutrients easily available to animals, which has also been used in some plant and animal protein sources [58–60]. Although the CM used in this experiment was not pretreated to break the cell wall, the high apparent digestibility of protein and lipid has proved its potential for application in shrimp feed. Both CAP and BPM are produced from bacteria fermentation and have attracted attention in the aquafeed field in the last decades. Our previous studies have demonstrated the availability of these two SCP in shrimp feed [61, 62]. In the present study, shrimp showed the highest digestibility to CAP, which may be due to the relatively pure composition of CAP. A previous study showed that the ADC_D_ and ADC_Pro_ of largemouth bass (*Micropterus salmoides*) increased with the higher dietary CAP and the activity of protease significantly increased in both the stomach and intestine [27]. In addition, the particle size and cell wall fragmentation of SCP may be one of the key factors affecting its application in aquafeeds [63]. The use of further ground and smaller particle size of BPM can replace a higher percentage of FM without affecting the growth performance of Japanese yellowtail (*Seriola quinqueradiata*) [23]. Further studies should be conducted in this aspect.

Plant-based proteins have been widely used in aquafeeds [64], but it is generally considered that they have disadvantages such as a lack of essential amino acids, rich in antinutritional factors, and poor palatability [37, 65]. Cottonseed meal is an inexpensive and highly practical protein source in shrimp feed. Usually, the cell walls of plant proteins are rich in crude fiber and ash and therefore difficult to be digested by shrimp. Previous studies demonstrated that the ADC_D_ of shrimp to cottonseed meal was about 50%-55% and ADC_Pro_ was about 57.6%-82.9% [66, 67]. Besides, gossypol is a natural terpenoid found in the glands of cotton and would reduce the intestinal nutrient digestion and absorption of bony fish [68], which is often mediated by triggering intestinal inflammation and disrupting the intestinal structure [69]. The low digestibility and toxic effects have become the main factors limiting the application of cottonseed meals in aquafeeds [70]. CPC is a high-quality protein produced from cottonseed meal after aqueous alcohol extraction to reduce soluble carbohydrates and remove most of the antinutritional factors [71]. The replacement of FM with 150 g/kg dietary CPC had no negative effects on the growth performance of the Pacific white shrimp, while the growth would be impaired with the further increase of substitution [67]. In the present study, although shrimp fed with dietary CPC showed lower apparent digestibility coefficients in all indices than those fed the CD, it was still better than the aforementioned results on cottonseed meal. On the other hand, even though CPC is abundant in phosphorus, it has mainly existed as the form of phytate phosphorus, which is difficult to be digested and absorbed by animals [72, 73] and leads to errors in the assay and insufficient samples. Pretreatment of cottonseed meal and other plant proteins with exogenous phytase or supplementation of the feed with phytase can effectively reduce the phytate phosphorus content and thus increase the availability of phosphorus and other micronutrients [74, 75]. Overall, the Pacific white shrimp showed good digestibility of diets and protein to CPC, but when using CPC to replace fishmeal in the feed, it is necessary to supplement the feed with an appropriate phosphorus source.

In conclusion, the apparent digestibility of six novel protein sources was evaluated in *Litopenaeus vannamei*. Results showed that shrimp had the highest apparent digestibility to SCP (CAP, BPM, and CM), followed by insect proteins (BSF and TM). Although the apparent digestibility of shrimp to the dietary CPC was lower than that of the other tested ingredients, it was better than that of the cottonseed meal. The six novel protein sources showed better digestive properties and appeared to be potential alternatives to fishmeal. This study will provide experimental evidence for the development of shrimp feeds containing novel protein sources.

## Figures and Tables

**Table 1:** Formulation of experimental diets (g/kg dry matter).

Ingredient	Diets	
	Control diet	Test diet
Brown fish meal	250.0	175.0
Soybean meal	250.0	175.0
Peanut meal	100.0	70.0
Wheat flour	240.6	168.3
Beer yeast	30.0	21.0
Shrimp shell meal	50.0	35.0
Fish oil	20.0	14.0
Soybean oil	20.0	14.0
Choline chloride	3.0	2.1
Soybean lecithin	10.0	7.0
Vitamin and mineral premix^a^	10.0	7.0
Calcium monophosphate	15.0	10.5
Vitamin C	1.0	0.7
Yttrium oxide	0.4	0.4
Testing ingredients		300.0
Total	1000.0	1000.0

^a^Vitamin and mineral premix (kg^−1^ of diet): thiamine, 5 mg; riboflavin, 10 mg; vitamin A, 5000 IU; vitamin E, 40 mg; vitamin D3, 1000 IU; menadione, 10 mg; pyridoxine, 10 mg; biotin, 0.1 mg; cyanocobalamin, 0.02 mg; calcium pantothenate, 20 mg; folic acid, 1 mg; niacin, 40 mg; vitamin C, 150 mg; iron, 100 mg; iodine, 0.8 mg; copper, 3 mg; zinc, 50 mg; manganese, 12 mg; selenium, 0.3 mg; cobalt, 0.2 mg.

**Table 2:** Nutrient level and amino acid composition of test ingredients (g/kg dry matter).

Index	FM	BSFLM	CM	CPC	TM	CAP	BPM
Nutrient level of ingredients							
Dry matter	932.0	911.0	937.9	947.5	915.7	928.6	954.0
Crude protein	682.1	351.7	515.3	615.1	658.8	842.1	741.0
Crude lipid	90.0	326.0	55.0	23.6	41.9	1.9	81.7
Amino acid composition of ingredients							
Methionine	18.0	6.5	9.0	8.5	12.9	22.9	17.3
Lysine	50.6	17.5	32.0	24.7	48.5	87.0	37.8
Leucine	45.4	21.3	42.4	34.4	50.8	63.8	50.4
Isoleucine	26.2	13.0	18.6	18.9	28.0	52.8	29.4
Histidine	20.2	10.6	12.9	18.0	9.0	16.8	14.2
Phenylalanine	26.3	14.5	28.2	35.3	25.7	33.0	29.1
Valine	31.0	20.2	29.5	26.6	39.2	54.4	38.9
Arginine	37.1	15.8	31.0	78.9	37.3	34.0	42.1
Threonine	29.0	14.8	25.7	19.0	24.6	40.2	28.7
Tyrosine	22.3	18.3	20.8	13.5	20.5	31.4	18.1
Aspartic acid	59.1	27.8	50.5	56.6	48.5	95.4	58.2
Serine	23.5	13.6	20.4	26.5	57.4	32.1	22.0
Glutamic acid	86.4	44.9	67.8	123.7	77.4	97.8	72.8
Glycine	38.2	17.1	27.3	25.0	53.1	38.7	33.3
Alanine	38.4	22.4	39.3	23.6	12.9	46.3	47.0
Proline	26.2	18.9	19.9	21.7	44.3	24.0	25.2
Cystine	6.5	4.4	5.8	9.5	40.5	7.1	3.5
Total	584.4	301.6	481.1	550.9	630.6	777.7	568.0

FM: fishmeal; BSFLM: black soldier fly larvae meal; CM: *Chlorella vulgaris* meal; CPC: cottonseed protein concentrate; TM: *Tenebrio molitor* meal; CAP: *Clostridium autoethanogenum* protein; BPM: methanotroph bacterial meal.

**Table 3:** Nutrient level and amino acid composition of experimental diets (g/kg dry matter).

Index	CD	BSFLM	CM	CPC	TM	CAP	BPM
Nutrient level of diets							
Dry matter	919.5	931.9	920.5	917.3	919.1	912.4	923.4
Crude protein	448.8	431.5	500.9	517.0	548.1	593.6	551.2
Crude lipid	71.8	141.5	75.0	52.1	65.0	48.7	67.0
Ash	106.4	101.6	90.8	100.6	104.1	86.5	93.0
Phosphorus	14.5	14.7	15.5	16.4	11.4	15.3	17.0
Amino acid composition of diets							
Methionine	6.8	6.4	7.9	8.0	9.3	12.2	11.3
Lysine	25.2	23.5	26.3	26.6	30.8	42.9	32.2
Leucine	31.4	29.7	35.8	34.3	37.1	44.8	39.9
Isoleucine	17.6	17.4	17.8	18.5	19.8	29.2	21.9
Histidine	11.3	10.4	9.7	13.7	8.9	10.6	11.2
Phenylalanine	19.4	17.7	24.5	27.1	25.1	29.9	27.1
Valine	20.7	22.4	21.2	21.5	24.5	28.5	24.3
Arginine	28.4	25.5	27.0	44.4	31.2	29.8	32.3
Threonine	16.0	15.9	17.1	16.6	18.8	24.9	20.8
Tyrosine	12.9	15.4	17.7	17.3	18.8	24.0	20.7
Aspartic acid	40.5	37.9	40.3	45.4	43.4	57.9	45.8
Serine	17.8	17.1	17.6	20.5	28.6	23.7	20.4
Glutamic acid	76.0	69.5	71.6	92.8	78.7	82.4	78.9
Glycine	21.3	21.0	22.9	23.4	30.2	27.7	25.5
Alanine	21.1	23.5	26.1	21.8	24.0	29.9	28.9
Proline	20.2	20.5	20.7	22.0	27.9	23.5	23.2
Cystine	4.8	4.6	5.2	5.2	5.6	6.3	5.1
Total	391.4	378.4	409.4	459.1	462.7	528.2	469.5

CD: control diet; BSFLM: black soldier fly larvae meal; CM: *Chlorella vulgaris* meal; CPC: cottonseed protein concentrate; TM: *Tenebrio molitor* meal; CAP: *Clostridium autoethanogenum* protein; BPM: methanotroph bacterial meal.

**Table 4:** Growth performance of the juvenile *Litopenaeus vannamei* fed with different diets.

Index	FBW	WGR	Survival	SGR	FE
CD	7.55 ± 0.13^c^	153.86 ± 6.69^c^	97.78 ± 1.11	2.33 ± 0.07^c^	1.72 ± 0.03^ab^
BSFLM	5.05 ± 0.02^a^	66.50 ± 2.18^a^	96.66 ± 3.33	1.27 ± 0.03^a^	2.83 ± 0.35^c^
CM	7.60 ± 0.10^c^	151.48 ± 3.34^c^	96.67 ± 0.00	2.31 ± 0.03^c^	1.73 ± 0.01^ab^
CPC	7.49 ± 0.05^c^	148.16 ± 4.90^c^	98.89 ± 1.11	2.27 ± 0.05^c^	1.72 ± 0.02^ab^
TM	6.35 ± 0.17^b^	105.54 ± 6.62^b^	96.66 ± 3.33	1.80 ± 0.08^b^	2.49 ± 0.02^bc^
CAP	7.81 ± 0.07^c^	155.52 ± 3.32^c^	93.33 ± 3.33	2.34 ± 0.03^c^	1.47 ± 0.12^a^
BPM	6.45 ± 0.08^b^	112.97 ± 3.24^b^	95.55 ± 2.93	1.89 ± 0.04^b^	2.11 ± 0.18^abc^

Data represent mean ± SEM of three replicates (*n* = 3). Values in the same line with different letters are significantly different (*P* < 0.05) based on Duncan's test. The lack of superscript letter indicates no significant differences among groups. FBW: final body weight (g); WGR: weight gain rate (%); SGR: specific growth rate (% day^−1^); FE: feed efficiency; CD: control diet; BSFLM: black soldier fly larvae meal; CM: *Chlorella vulgaris* meal; CPC: cottonseed protein concentrate; TM: *Tenebrio molitor* meal; CAP: *Clostridium autoethanogenum* protein; BPM: methanotroph bacterial meal.

**Table 5:** Apparent digestibility coefficients for dry matter of diets and ingredients, crude protein, crude lipid, and phosphorus (%) of test ingredients in *Litopenaeus vannamei.*

Index	ADC_D_	ADC_I_	ADC_Pro_	ADC_L_	ADC_P_
CD	79.27 ± 0.77^e^		91.67 ± 0.31^e^	94.05 ± 0.26^d^	50.49 ± 5.88^b^
BSFLM	71.19 ± 0.64^ab^	44.27 ± 2.80^a^	72.41 ± 0.47^b^	86.65 ± 0.14^c^	41.40 ± 2.13^a^
CM	75.19 ± 1.11^cd^	61.58 ± 4.82^b^	83.80 ± 0.64^c^	85.74 ± 0.62^c^	76.03 ± 1.07^c^
CPC	67.52 ± 3.81^a^	39.67 ± 5.12^a^	83.44 ± 0.29^c^	70.53 ± 3.07^a^	Error
TM	71.71 ± 0.58^bc^	46.49 ± 2.51^a^	56.50 ± 0.76^a^	80.33 ± 1.51^b^	45.68 ± 1.25^ab^
CAP	83.46 ± 0.37^f^	97.41 ± 1.62^c^	97.74 ± 0.15^f^	93.58 ± 0.57^d^	90.95 ± 0.49^d^
BPM	76.62 ± 1.91^de^	67.79 ± 8.28^b^	87.22 ± 0.34^d^	91.66 ± 0.45^d^	69.23 ± 0.27^c^

Data represents mean ± SEM of three replicates (*n* = 3). Values in the same line with different letters are significantly different (*P* < 0.05) based on Duncan's test. The lack of superscript letter indicates no significant differences among groups, where apparent digestibility coefficient of dry matter (ADC_D_), ingredients (ADC_I_), crude protein (ADC_Pro_), crude lipid (ADC_L_), and phosphorus (ADC_P_); CD: control diet; BSFLM: black soldier fly larvae meal; CM: *Chlorella vulgaris* meal; CPC: cottonseed protein concentrate; TM: *Tenebrio molitor* meal; CAP: *Clostridium autoethanogenum* protein; BPM: methanotroph bacterial meal. Error: the ADC_P_ for CPC could not be analyzed due to insufficient samples, so the corresponding data are not provided.

**Table 6:** Apparent digestibility coefficients for amino acids of test ingredients in *Litopenaeus vannamei* (%).

Index	CD	BSFLM	CM	CPC	TM	CAP	BPM
Essential amino acid							
Lysine	94.78	83.08	87.11	78.18	85.91	98.26	95.79
Methionine	87.50	69.34	87.32	87.84	87.56	98.21	98.97
Arginine	95.91	80.47	90.43	93.27	59.43	96.92	92.37
Histidine	92.48	62.69	74.50	87.65	55.20	86.94	79.38
Valine	93.74	74.63	81.70	80.53	46.37	95.20	84.59
Threonine	92.03	73.20	85.60	78.24	50.72	97.28	88.90
Phenylalanine	92.36	73.88	86.28	88.46	57.43	95.23	87.63
Isoleucine	93.82	75.47	82.35	79.74	54.44	97.00	87.27
Leucine	93.99	75.88	86.75	82.86	54.66	98.05	89.61
Nonessential amino acid							
Aspartic acid	93.19	76.67	86.22	86.35	58.29	97.70	89.02
Serine	92.43	68.83	86.09	85.83	55.44	96.99	88.81
Glutamic acid	94.97	80.49	88.92	91.50	62.14	96.81	89.05
Glycine	89.73	59.38	83.85	80.60	55.93	96.48	87.33
Alanine	92.44	75.47	87.10	76.01	25.25	96.51	86.06
Cystine	92.01	69.28	81.86	80.16	74.16	98.05	81.29
Tyrosine	91.73	77.01	87.96	89.35	58.85	95.42	91.36
Proline	93.69	70.58	90.12	88.42	52.72	95.97	94.74

CD: control diet; BSFLM: black soldier fly larvae meal; CM: *Chlorella vulgaris* meal; CPC: cottonseed protein concentrate; TM: *Tenebrio molitor* meal; CAP: *Clostridium autoethanogenum* protein; BPM: methanotroph bacterial meal.

## Data Availability

The data that support the findings of this study are available on request from the corresponding author.

## References

[1] Cummins V. C., Rawles S. D., Thompson K. R. (2017). Evaluation of black soldier fly (*Hermetia illucens*) larvae meal as partial or total replacement of marine fish meal in practical diets for Pacific white shrimp (*Litopenaeus vannamei*). *Aquaculture*.

[2] Amaya E., Davis D. A., Rouse D. B. (2007). Alternative diets for the Pacific white shrimp _Litopenaeus vannamei_. *Aquaculture*.

[3] Gui L., Mai H., Chi S., Zhou W., Li Y., Tan B. (2019). Effects of yeast culture on growth performance, hematological parameters, immunity and disease resistance of Litopenaeus vannamei. *Journal of Guangdong Ocean University*.

[4] Food and Agriculture Organization of the United Nations (2020). *The state of world fisheries and aquaculture 2018*.

[5] Li W., Li L., Liu H. Y. (2022). Effects of Clostridium butyricum on growth, antioxidant capacity and non-specific immunology of Litopenaeus vannamei fed with concentrated cottonseed protein replacement of fishmeal. *Journal of Guangdong Ocean University*.

[6] Wang G., Sun Y., Niu F. (2017). Effects of exogenous enzyme supplementation on digestive enzyme activity, apparent digestibility and fecal nitrogen and phosphorus content of juvenile yellow catfish. *Journal of Guangdong Ocean University*.

[7] Wirtz A., Carter C. G., Codabaccus M. B., Fitzgibbon Q. P., Townsend A. T., Smith G. G. (2022). Protein sources influence both apparent digestibility and gastrointestinal evacuation rate in juvenile slipper lobster (Thenus australiensis). *Comparative Biochemistry and Physiology Part A: Molecular & Integrative Physiology*.

[8] Van Huis A., Van Itterbeeck J., Klunder H. (2013). *Edible insects: future prospects for food and feed security no. 171*.

[9] Makkar H. P. S., Tran G., Heuzé V., Ankers P. (2014). State-of-the-art on use of insects as animal feed. *Animal Feed Science and Technology*.

[10] Xie Y., Peng K., Hu J., Wang G. (2022). Review on application of black soldier fly (Hermetia illucens linnaeus) in aquatic feed. *Journal of Guangdong Ocean University*.

[11] Dumas A., Raggi T., Barkhouse J., Lewis E., Weltzien E. (2018). The oil fraction and partially defatted meal of black soldier fly larvae (*Hermetia illucens*) affect differently growth performance, feed efficiency, nutrient deposition, blood glucose and lipid digestibility of rainbow trout (*Oncorhynchus mykiss*). *Aquaculture*.

[12] Lu R., Chen Y., Yu W. (2020). Defatted black soldier fly (*Hermetia illucens*) larvae meal can replace soybean meal in juvenile grass carp (*Ctenopharyngodon idellus*) diets. *Aquaculture Reports*.

[13] Bordiean A., Krzyżaniak M., Stolarski M. J., Czachorowski S., Peni D. (2020). Will yellow mealworm become a source of safe proteins for Europe?. *Agriculture*.

[14] Gasco L., Henry M., Piccolo G. (2016). *Tenebrio molitor* meal in diets for European sea bass (*Dicentrarchus labrax*L.) juveniles: growth performance, whole body composition and *in vivo* apparent digestibility. *Animal Feed Science and Technology*.

[15] Piccolo G., Iaconisi V., Marono S. (2017). Effect of *Tenebrio molitor* larvae meal on growth performance, *in vivo* nutrients digestibility, somatic and marketable indexes of gilthead sea bream (*Sparus aurata*). *Animal Feed Science and Technology*.

[16] Saeed M., Yasmin I., Murtaza M. A., Fatima I., Saeed S. (2016). Single cell proteins: a novel value added food product. *Pakistan Journal of Food Sciences*.

[17] Ma G., Xu P., Song L., Zhang Y. (2014). Research progress of ecological factors affecting the synthesis of EPA in microalgae. *Journal of Guangdong Ocean University*.

[18] Teimouri M., Yeganeh S., Mianji G. R., Najafi M., Mahjoub S. (2019). The effect of spirulina platensis meal on antioxidant gene expression, total antioxidant capacity, and lipid peroxidation of rainbow trout (Oncorhynchus mykiss). *Fish Physiology and Biochemistry*.

[19] Raji A. A., Jimoh W. A., Bakar N. (2020). Dietary use of spirulina (Arthrospira) and chlorella instead of fish meal on growth and digestibility of nutrients, amino acids and fatty acids by African catfish. *Journal of Applied Phycology*.

[20] Tibbetts S. M., Mann J., Dumas A. (2017). Apparent digestibility of nutrients, energy, essential amino acids and fatty acids of juvenile Atlantic salmon (*Salmo salar* L.) diets containing whole- cell or cell-ruptured *Chlorella vulgaris* meals at five dietary inclusion levels. *Aquaculture*.

[21] Jones S. W., Karpol A., Friedman S., Maru B. T., Tracy B. P. (2020). Recent advances in single cell protein use as a feed ingredient in aquaculture. *Current Opinion in Biotechnology*.

[22] Chen Y., Chi S., Zhang S. (2021). Replacement of fish meal with Methanotroph (*Methylococcus capsulatus* , Bath) bacteria meal in the diets of Pacific white shrimp (*Litopenaeus vannamei*). *Aquaculture*.

[23] Biswas A., Takakuwa F., Yamada S. (2020). Methanotroph (*Methylococcus capsulatus* , Bath) bacteria meal as an alternative protein source for Japanese yellowtail, *Seriola quinqueradiata*. *Aquaculture*.

[24] Zheng J., Zhang W., Dan Z. (2023). Effects of fish meal replaced by methanotroph bacteria meal (Methylococcus capsulatus) on growth, body composition, antioxidant capacity, amino acids transporters and protein metabolism of turbot juveniles (Scophthalmus maximus L.). *Aquaculture*.

[25] Wei H., Yu H., Chen X. (2018). Effects of soybean meal replaced by Clostridium autoethanogenum protein on growth performance, plasma biochemical indexes and hepatopancreas and intestinal histopathology of grass carp (Ctenopharyngodon idllus). *Chinese Journal of Animal Nutrition*.

[26] Yao W., Yang P., Zhang X. (2022). Effects of replacing dietary fish meal with *Clostridium autoethanogenum* protein on growth and flesh quality of Pacific white shrimp (*Litopenaeus vannamei*). *Aquaculture*.

[27] Zhu S., Gao W., Wen Z. (2022). Partial substitution of fish meal by *Clostridium autoethanogenum* protein in the diets of juvenile largemouth bass (*Micropterus salmoides*). *Aquaculture Reports*.

[28] Cui X., Ma Q., Duan M., Xu H., Liang M., Wei Y. (2022). Effects of fishmeal replacement by *Clostridium autoethanogenum* protein on the growth, digestibility, serum free amino acid and gene expression related to protein metabolism of obscure pufferfish (*Takifugu obscurus*). *Animal Feed Science and Technology*.

[29] Ye G., Dong X., Yang Q. (2020). Low-gossypol cottonseed protein concentrate used as a replacement of fish meal for juvenile hybrid grouper (Epinephelus fuscoguttatus ♀ × Epinephelus lanceolatus ♂): Effects on growth performance, immune responses and intestinal microbiota. *Aquaculture*.

[30] Zhang H., Pu X., Yang Q. (2018). Effects of replacing fish meal with high-protein cottonseed meal on growth performance, non-specific immune index and disease resistance for Litopenaeus vannamei. *Journal of Guangdong Ocean University*.

[31] National Research Council (2011). *Nutrient requirements of fish and shrimp*.

[32] Association of Official Analytical Chemists (2006). *Official Methods of Analysis*.

[33] Wu Y. C., Li R. M., Shen G. R. (2021). Effects of dietary small peptides on growth, antioxidant capacity, nonspecific immunity and ingut microflora structure of Litopenaeus vannamei. *Journal of Guangdong Ocean University*.

[34] Pan J., Han Y., Huo Y., Su P., Jiang Z. (2016). Effects of dietary alginate oligosaccharide on intestinal morphology, activities of digestive enzymes and apparent digestibility of turbot (Scophthalmus maximus l). *Journal of Guangdong Ocean University*.

[35] Yang Q., Zhou X., Zhou Q., Tan B., Chi S., Dong X. (2009). Apparent digestibility of selected feed ingredients for white shrimp Litopenaeus vannamei, Boone. *Aquaculture Research*.

[36] Jannathulla R., Dayal J. S., Vasanthakumar D., Ambasankar K., Muralidhar M. (2018). Effect of fungal fermentation on apparent digestibility coefficient for dry matter, crude protein and amino acids of various plant protein sources in Penaeus vannamei. *Aquaculture Nutrition*.

[37] Hua K., Cobcroft J. M., Cole A. (2019). The future of aquatic protein: implications for protein sources in aquaculture diets. *One Earth*.

[38] Meyer-Rochow V. B., Gahukar R. T., Ghosh S., Jung C. (2021). Chemical composition, nutrient quality and acceptability of edible insects are affected by species, developmental stage, gender, diet, and processing method. *Food*.

[39] Woods M., Goosen N., Hoffman L., Pieterse E. (2020). A simple and rapid protocol for measuring the chitin content ofHermetia illucens(L.) (Diptera: Stratiomyidae) larvae. *Journal of Insects as Food and Feed*.

[40] Song Y., Kim M., Moon C. (2018). Extraction of chitin and chitosan from larval exuvium and whole body of edible mealworm, Tenebrio molitor. *Entomological Research*.

[41] Melenchón F., Larrán A. M., De Mercado E. (2021). Potential use of black soldier fly (Hermetia illucens) and mealworm (Tenebrio molitor) insectmeals in diets for rainbow trout (Oncorhynchus mykiss). *Aquaculture Nutrition*.

[42] Shin J., Lee K. (2021). Digestibility of insect meals for pacific white shrimp (Litopenaeus vannamei) and their performance for growth, feed utilization and immune responses. *PLoS One*.

[43] Panini R. L., Freitas L. E. L., Guimarães A. M. (2017). Potential use of mealworms as an alternative protein source for pacific white shrimp: digestibility and performance. *Aquaculture*.

[44] Abun T. W., Haetami K. (2016). Effect of time processing at steps of bioprocess shrimp waste by three microbes on protein digestibility and metabolizable energy products of native chicken. *Agrolife Scientific Journal*.

[45] Cheng A., Shiu Y., Chiu S., Ballantyne R., Liu C. H. (2021). Effects of chitin from _Daphnia similis_ and its derivative, chitosan on the immune response and disease resistance of white shrimp, _Litopenaeus vannamei_. *Fish & Shellfish Immunology*.

[46] Turck D., Castenmiller J., De Henauw S. (2021). Safety of dried yellow mealworm (Tenebrio molitor larva) as a novel food pursuant to regulation (eu) 2015/2283. *EFSA Journal*.

[47] Cullere M., Tasoniero G., Giaccone V. (2016). Black soldier fly as dietary protein source for broiler quails: apparent digestibility, excreta microbial load, feed choice, performance, carcass and meat traits. *Animal*.

[48] Sankian Z., Khosravi S., Kim Y., Lee S. M. (2018). Effects of dietary inclusion of yellow mealworm (*Tenebrio molitor*) meal on growth performance, feed utilization, body composition, plasma biochemical indices, selected immune parameters and antioxidant enzyme activities of mandarin fish (*Siniperca scherzeri*) juveniles. *Aquaculture*.

[49] Khosravi S., Kim E., Lee Y., Lee S. M. (2018). Dietary inclusion of mealworm (Tenebrio molitor) meal as an alternative protein source in practical diets for juvenile rockfish (Sebastes schlegeli). *Entomological Research*.

[50] Motte C., Rios A., Lefebvre T., do H., Henry M., Jintasataporn O. (2019). Replacing fish meal with defatted insect meal (yellow mealworm Tenebrio molitor) improves the growth and immunity of pacific white shrimp (Litopenaeus vannamei). *Animals*.

[51] Chen Y., Chi S., Zhang S. (2021). Effect of black soldier fly (Hermetia illucens) larvae meal on lipid and glucose metabolism of pacific white shrimp Litopenaeus vannamei. *British Journal of Nutrition*.

[52] Errico S., Spagnoletta A., Verardi A., Moliterni S., Dimatteo S., Sangiorgio P. (2022). Tenebrio molitor as a source of interesting natural compounds, their recovery processes, biological effects, and safety aspects. *Comprehensive Reviews in Food Science and Food Safety*.

[53] Sharif M., Zafar M. H., Aqib A. I., Saeed M., Farag M. R., Alagawany M. (2021). Single cell protein: sources, mechanism of production, nutritional value and its uses in aquaculture nutrition. *Aquaculture*.

[54] Cao S., Zou T., Zhang P. (2018). Effects of dietary fishmeal replacement with Spirulina platensis on the growth, feed utilization, digestion and physiological parameters in juvenile Gibel carp (Carassis auratus gibelio var. Cas iii). *Aquaculture Research*.

[55] Zhang C., Lao Z., Liu Y. (2007). Change of phytoplankton and physicochemical factors in ponds of shrimp Penaeus vannamei with different cultural patterns during late period. *Journal of Guangdong Ocean University*.

[56] Pakravan S., Akbarzadeh A., Sajjadi M., Hajimoradloo A., Noori F. (2018). Chlorella vulgaris meal improved growth performance, digestive enzyme activities, fatty acid composition and tolerance of hypoxia and ammonia stress in juvenile pacific white shrimp Litopenaeus vannamei. *Aquaculture Nutrition*.

[57] Maliwat G. C., Velasquez S., Robil J. L. (2017). Growth and immune response of giant freshwater prawn Macrobrachium rosenbergii (de man) postlarvae fed diets containing Chlorella vulgaris (beijerinck). *Aquaculture Research*.

[58] Sánchez-Muros M. J., Renteria P., Vizcaino A., Barroso F. G. (2020). Innovative protein sources in shrimp (Litopenaeus vannamei) feeding. *Reviews in Aquaculture*.

[59] Zhu X., Deng Q., Guo H., Li G., Zhu C. (2021). Effects of dietary hydrolyzable tannins on growth performance, antioxidant capacity, intestinal microflora and resistance against Vibrio parahaemolyticus of juvenile Pacific white shrimp, litopenaeus vannamei (Boone, 1931). *Journal of Guangdong Ocean University*.

[60] Wang A., Yang Q., Tan B. (2018). Effects of enzymolytic soybean meal on growth performance, serum biochemical indices, non-specific immunity and disease resistance of juvenile Litopenaeus vannamei. *Journal of Guangdong Ocean University*.

[61] Chen Y., Chi S., Zhang S. (2022). Evaluation of methanotroph (Methylococcus capsulatus, bath) bacteria meal on body composition, lipid metabolism, protein synthesis and muscle metabolites of pacific white shrimp (Litopenaeus vannamei). *Aquaculture*.

[62] Zheng C., Gong S., Cao J. (2022). Effects of dietary lipid sources on alleviating the negative impacts induced by the fishmeal replacement with Clostridium autoethanogenum protein in the diet of pacific white shrimp (Litopenaeus vannamei). *Science*.

[63] Chama M. K. H., Liang H., Huang D. (2021). Methanotroph (*Methylococcus capsulatus*, Bath) as an alternative protein source for genetically improved farmed tilapia (GIFT: *Oreochromis niloticus*) and its effect on antioxidants and immune response. *Aquaculture Reports*.

[64] Mo X., Huang C., Jiang S. (2021). Effects of concentrated dephenolic cottonseed protein instead of fish meal on stress resistance of Penaeus monodon. *The Israeli Journal of Aquaculture-Bamidgeh*.

[65] Zhang W., Tan B., Pang A., Deng J., Yang Q., Zhang H. (2021). Screening of potential biomarkers for soybean meal induced enteritis in pearl gentian grouper (Espinephelus fuscoguttatus♀× Epinephelus lanceolatus♂). *Journal of Guangdong Ocean University*.

[66] Siccardi A. J., Richardson C. M., Dowd M. K., Wedegaertner T. C., Samocha T. M. (2016). Digestibility of glandless cottonseed protein in diets for pacific white shrimp, Litopenaeus vannamei. *Journal of the World Aquaculture Society*.

[67] Wan M., Yin P., Fang W. (2018). The effect of replacement of fishmeal by concentrated dephenolization cottonseed protein on the growth, body composition, haemolymph indexes and haematological enzyme activities of the pacific white shrimp (Litopenaeus vannamei). *Aquaculture Nutrition*.

[68] Wang K., Jiang W., Wu P. (2018). Gossypol reduced the intestinal amino acid absorption capacity of young grass carp (*Ctenopharyngodon idella*). *Aquaculture*.

[69] Liu H., Dong X., Tan B. (2020). Effects of fish meal replacement by low-gossypol cottonseed meal on growth performance, digestive enzyme activity, intestine histology and inflammatory gene expression of silver sillago (Sillago sihama Forsskál) (1775). *Aquaculture Nutrition*.

[70] Wang J., Zhang H., Yang Q. (2020). Effects of replacing soybean meal with cottonseed meal on growth, feed utilization and non-specific immune enzyme activities for juvenile white shrimp, _Litopenaeus vannamei_. *Aquaculture Reports*.

[71] Yin B., Liu H., Tan B. (2018). Cottonseed protein concentrate (CPC) suppresses immune function in different intestinal segments of hybrid grouper _♀ Epinephelus fuscoguttatus_× _♂ Epinephelus lanceolatu_ via TLR-2/MyD88 signaling pathways. *Fish & Shellfish Immunology*.

[72] Lemos D., Tacon A. G. (2017). Use of phytases in fish and shrimp feeds: a review. *Reviews in Aquaculture*.

[73] An W., Chen X., Li W., Dong X., Tan B., Zhao X. (2018). Optimum calcium and phosphorus supplemental levels in diets of large size Litopenaeus vannamei. *Journal of Guangdong Ocean University*.

[74] von Danwitz A., van Bussel C. G., Klatt S. F., Schulz C. (2016). Dietary phytase supplementation in rapeseed protein based diets influences growth performance, digestibility and nutrient utilisation in turbot (*Psetta maxima* L.). *Aquaculture*.

[75] Li L., Zhang D., Su T., Gong S., Jiang S. (2007). Isolation and identification of a phytase-producing strain. *Journal of Guangdong Ocean University*.

